# Patient-reported symptoms and diagnostic journey in Multiple Myeloma

**DOI:** 10.3389/fonc.2023.1282569

**Published:** 2023-11-30

**Authors:** Rohit Vijjhalwar, Kaiyang Song, Roshi Shrestha, Stella Bowcock, Maria T. Sanchez-Santos, Karthik Ramasamy, Muhammad Kassim Javaid

**Affiliations:** ^1^ Medical Sciences Division, University of Oxford, Oxford, United Kingdom; ^2^ Nuffield Department of Orthopaedics, Rheumatology and Musculoskeletal Sciences, Oxford, United Kingdom; ^3^ Department of Haematology, King's College Hospital NHS Trust, London, United Kingdom; ^4^ Department of Clinical Haematology, Oxford University Hospitals, NHS Foundation Trust, Oxford, United Kingdom

**Keywords:** myeloma, diagnostic delay, digital platforms, dynamic consent, oncology

## Abstract

**Introduction:**

Late presentation of multiple myeloma (MM) heightens the risk of complication risks, including end-organ damage. This study aimed to: 1) detail the diagnostic journey of MM patients, encompassing symptoms, initial diagnoses, and healthcare professionals met; 2) establish the median duration from symptom onset to MM diagnosis; and 3) examine factors linked to timely MM diagnosis within 12 weeks.

**Methods:**

A total of 300 adults self-reporting MM were analysed from the Rare and Undiagnosed Diseases cohort Study (RUDY). The RUDY study is a web-based platform, where participants provide dynamic consent and self-report their MM diagnosis and information about their diagnostic journey. This includes the estimated date of initial potential first symptoms, descriptions of these symptoms, the healthcare professionals they consulted, and other diagnoses received before the MM diagnosis. Descriptive statistics, combinatorial analyses and logistic regression analyses were used to describe and examine the diagnostic journey of individuals with MM.

**Results:**

Overall, 52% of the participants reported other diagnoses before MM diagnosis, with musculoskeletal disorders (47.8%), such as osteoporosis, costochondritis, or muscle strains, being the most common. The most prevalent initial reported symptom was back pain/vertebral fractures (47%), followed by chest/shoulder pain, including rib pain and fractures (20%), and fatigue/tiredness (19.7%). 40% of participants were diagnosed by direct referral from primary care to haematology without seeing other healthcare professionals whilst 60% consulted additional specialists before diagnosis. The median time from symptom onset to MM diagnosis was 4 months (IQR 2-10 months, range 0-172). Seeing an Allied Healthcare Professional such as a physiotherapist, chiropractor or an osteopath (OR = 0.25, 95% CI [0.12, 0.47], p <0.001), experiencing infection symptoms (OR = 0.32, 95% CI [0.13, 0.76], p = 0.013), and having chest or shoulder pain (OR = 0.45, 95% CI [0.23, 0.86], p = 0.020) were associated with a lower likelihood of being diagnosed with MM within 12 weeks. Older age (OR = 1.04, 95% CI [1.02, 1.07], p = 0.001) was associated with a higher likelihood of diagnosis within 12 weeks.

**Discussion:**

Developing resources for allied health professionals may improve early recognition of MM.

## Introduction

Multiple Myeloma (MM) is a hematologic malignancy characterised by abnormal clonal plasma cell infiltration in the bone marrow. (1) In the United Kingdom (UK), the annual incidence of MM is 6,000 cases and accounts for around 3,100 deaths annually. (2, 3) This can be due to end-organ damage associated with the disease including haematological issues (such as anemia, bone marrow failure, and poor immunity) (4), bone-related complications (including pathological fractures, lytic bone lesions, and hypercalcemia) (5), renal insufficiency (6), and neurological complications (such as compression of the spinal cord, nerve roots, and peripheral neuropathy (7).

Timely and accurate diagnosis of MM has been linked to higher survival rates at the 1-year and 5-year marks. (8, 9) Conversely, delays in diagnosis may be associated with end-organ damage and a larger disease burden for patients. (9–12) Additionally, receiving a diagnosis through an emergency pathway is linked to worse prognoses, and end-organ damage, (13) compared to obtaining a diagnosis directly referred between primary and secondary care. (14) Hence, early detection is a high priority for patients. Despite this, studies have shown that patients with MM have one of the longest time-to-diagnosis intervals, of all cancers, with an average time between symptom onset and MM diagnosis of 99 days. (15–17) Existing standards in the Faster diagnosis framework (FDS) for cancer diagnosis in the UK state that patients must have cancer ruled out or receive a diagnosis within 28 days from GP referral to secondary care. (18) Additionally, once initially referred for suspected cancer, patients should begin treatment within 62 days (18).

The Haematological Malignancy Research Network (HMRN) in the UK has highlighted the issue of potential delays in diagnosing MM in primary care due to the presence of non-specific symptoms. (17, 19, 20) In this study, we examined data obtained from the Rare and Undiagnosed Disease Study (RUDY study), an ongoing prospective cohort study based in the UK, with the aim of examining the diagnostic journey for adults diagnosed with MM. (21) Our analysis focuses on first symptoms and range of healthcare professionals consulted and their association with a timely diagnosis of MM within 12 weeks.

## Methods

### Study population

Data from the Rare and Undiagnosed Diseases Study (RUDY study), an ongoing UK-based multi-centre prospective cohort study launched in 2014, aimed at understanding the impact of rare diseases was used (LREC 14/SC/0126 & RUDY LREC 17/SC/0501. (21) The RUDY study is conducted through a web-based platform, where participants provide dynamic consent and self-report their MM diagnosis and information about their diagnostic journey. This includes the estimated date of initial potential first symptoms, descriptions of these symptoms, the healthcare professionals they consulted, and other diagnoses received before the MM diagnosis. We included all participants who self-reported having MM on this platform, including those who had been diagnosed before 2014. The RUDY study questionnaire is included as a Supplementary questionnaire.

RUDY is accessed through www.rudystudy.org and promoted to patients with MM by Myeloma UK, as well as social media such as Twitter. The diagnostic journey was reported by the participants in the RUDY platform as part of the diagnostic form based on the EPIRARE recommendations. (22) Only participants aged 16 years and over with a self-reported diagnosis of MM were included.

Patients who had prior diagnoses of Monoclonal gammopathy of undetermined significance (MGUS) or smouldering myeloma were not included in the analysis due to the unavailability of recorded diagnostic dates for these pre-myeloma conditions.

### Outcomes

The primary outcome was achieving a time from the first potential symptom to the diagnosis of multiple myeloma (MM) within 12 weeks, based on UK standards and clinical expertise (SB, KR). This is based on UK standards outlined by the National Institute for Health and Care Excellence (NICE) and the British Committee for Standards in Haematology (BCSH). (23, 24) These standards aim to improve care for people with MM by promoting the most effective tests and treatments for MM and its complications. These time frames are crucial in ensuring timely diagnosis and treatment of MM. The application of these standards in this study was guided by clinical expertise (SB, KR).

The approach aligns with Anderson’s model of total delay in cancer diagnosis. (25) In this model, appraisal delay represents the time taken for a participant to recognise symptoms. Illness delay is the time taken for a participant to decide to seek medical help after recognising illness. Behavioural delay is the time taken for a participant to act on the decision to seek medical help. Finally, scheduling delay is the time taken for a participant to be seen by the primary and secondary care teams. Based on clinical expertise, we allowed for a 6-week patient appraisal (for the participant to recognise symptoms) and primary care intervals (for the participant to consult with GP), 2 weeks to hospital appointment and 4 weeks to diagnosis. (25) Similarly, a previous study that focused on MM found that the appraisal interval was between 2 weeks to 7 months. (26) This adaptation of Anderson’s model takes into account real-world constraints and variations in patient behaviour and healthcare system responses. It provides a pragmatic approach that aligns with UK standards while also considering individual patient circumstances.

Additionally, secondary outcomes included examining the various healthcare professionals consulted before the MM diagnosis, the symptoms presented to the general practitioner (GP), and other diagnoses received before the MM diagnosis.

### Symptoms and medical professionals

Symptom groups were categorised into back pain (including vertebral fractures), chest and shoulder pain (including rib fractures), pelvic and leg pain, non-specific pain, infection, fatigue or tiredness, kidney disease, anaemia, shortness of breath, abdominal discomfort, other fractures and other symptoms. Infection was a broad group describing a wide set of infections including upper- and lower-respiratory tract infections, urinary tract infections, epiglottitis, skin infections etc. Symptom groups were non-exclusive hence each patient-reported symptom descriptions may be grouped into multiple groups. The decision to have separate categories for different localisations of pain and fractures in this study was made to gain a more nuanced understanding of the symptoms that the patients presented with to primary care. ‘Other symptoms’ were used to group all symptoms that were vague symptoms reported by patients which could not be accurately categorised into symptoms groups. It was not included in the combinatorial analysis.

A diverse range of medical specialisations were reported, including Allied healthcare professionals (such as Chiropractors, Osteopaths and Physiotherapists), Orthopaedics, A&E physicians, Oncologists, Rheumatology, Renal medicine, Gastroenterology, Respiratory, Neurology or Neurosurgery, Cardiology, ENT, Ophthalmology, Urology, Pain clinic, General Surgery, Endocrinology, Maxillofacial surgery, Dermatologist, Obstetrics and gynaecology, and Infectious disease. Allied health professionals (AHPs) was a collective term used to describe Physiotherapists, Osteopaths, and Chiropractors for the purpose of analysis.

### Predictors

In our logistic regression analysis, we considered a range of predictors that could be associated with a diagnosis of MM within 12 weeks. This included healthcare professionals seen during their diagnostic journey as well as their initial reported symptoms. Only predictors that were reported by more than 5% of the patients were selected for univariate and multivariate logistic regression analysis. The age at which they reported their first symptom and sex were considered as key predictors to include in the analysis.

### Diagnoses received prior to MM diagnosis

Diagnoses patients received prior to MM diagnosis were also reported. This was coded into eleven separate categories of pain disorders, musculoskeletal (MSK), neurological, infective, other haematological cancers, non-haematological cancer, MGUS, gastrointestinal, renal, vitamin or electrolyte imbalances or other disorders. These were not included in the predictive model or combinatorial analysis.

### Data analysis

Variables were checked for normality. Descriptive statistics were employed to summarise patient characteristics and disease-related factors. Combinatorial analyses were used to achieve precise categorisation of patient symptom descriptions and healthcare professionals seen using R packages UpSetR (analysis) (27) and ggupset (visualisation). In addition, we conducted an analysis of the healthcare professionals consulted by the participants during their diagnostic journey. 26 patients self-reported the names of doctors rather than their speciality. As we could not elucidate their speciality, these were excluded from the analysis. Regarding the multivariate logistic regression, the linearity of continuous variables with the outcome was assessed using fractional polynomials and collinearity between variables was assessed by the variance inflation factor (VIF). The final logistic multivariate regression model for predicting diagnosis within 12 weeks was determined through stepwise logistic regressions with backward elimination using R packages caret and MASS.

All statistical analysis was performed in R using RStudio (Version 2022.07.2).

## Results

### Cohort characteristics

In total, 617 participants who self-reported MM were recruited into the RUDY study. Diagnostic pathways were available for 318 participants; the remaining participants had not recorded enough information to determine their diagnostic pathway and were excluded from the study. Of the 318 participants, 18 participants were excluded due to a prior diagnosis of MGUS or smouldering myeloma due to the unavailability of recorded diagnostic dates for these pre-myeloma conditions. Sensitivity analysis was performed to ensure the exclusion of the patients did not affect the Overall, the median age when initial symptoms first appeared was 59 years (Interquartile range [IQR]= 53, 66), with the final cohort comprising 59% males and 41% females. **Table 1** provides a summary of the final cohort’s demographic characteristics, split by gender.

**Table 1:** Cohort demographics.

Study population characteristics stratified by type of gender	F, N = 123^1^	M, N = 177^1^	Whole cohort, N=300^1^
**Age at inclusion in RUDY**	65 (59,70)	69 (61, 74)	67 (60, 74)
Age Categories			
Under 40	2 (1.6%)	0 (0%)	2 (0.7%)
40-49	10 (8.1%)	7 (4.0%)	17 (5.7%)
50-59	22 (18%)	30 (17%)	52 (17.3%)
60-69	54 (44%)	55 (31%)	109 (36.3%)
70-79	28 (23%)	70 (40%)	98 (32.7%)
80+	7 (5.7%)	15 (8.5%)	22 (7.3%)
**Age at first symptom**	57 (52, 62)	61 (55, 68)	59 (53, 66)

^1^Median (IQR); n (%).

### Interim diagnoses given

Before being diagnosed with MM, over half of the patients (52%, 157 patients) received other diagnoses while 48% of patients (n=143) did not receive any interim diagnoses before their final MM diagnosis. Among the interim diagnoses, musculoskeletal disorders (47.8%) were the most prevalent including conditions like osteoporosis, costochondritis, or muscle strains. Interestingly, in a small group of patients, another haematological cancer was suspected (3.2%, n=5) before their eventual MM diagnosis. **Table 2** summarises the interim diagnoses commonly given to MM patients before their final MM diagnosis.

**Table 2:** Other diagnoses given prior to myeloma diagnosis.

Diagnoses given	N = 300^1^
No interim diagnoses given	143 (48%)
**Interim diagnoses given**	**N = 157 (52% of whole cohort)** ^1^
Musculoskeletal pathology	75 (47.8%)
Other diagnoses	25 (15.9%)
Unspecified pain syndrome	22 (14.0%)
Infection	17 (10.8%)
Neurological pathology	15 (9.6%)
Kidney disease	13 (8.3%)
Gastrointestinal disease	8 (5.1%)
Non-haematological cancer	6 (3.8%)
Haematological cancer	5 (3.2%)
Vitamin or electrolyte imbalance	4 (2.5%)

^1^n (%).

As more than one other diagnosis could have been self-reported, the percentage totals exceed 100%.

### Initial presenting symptoms

**Figure 1:**
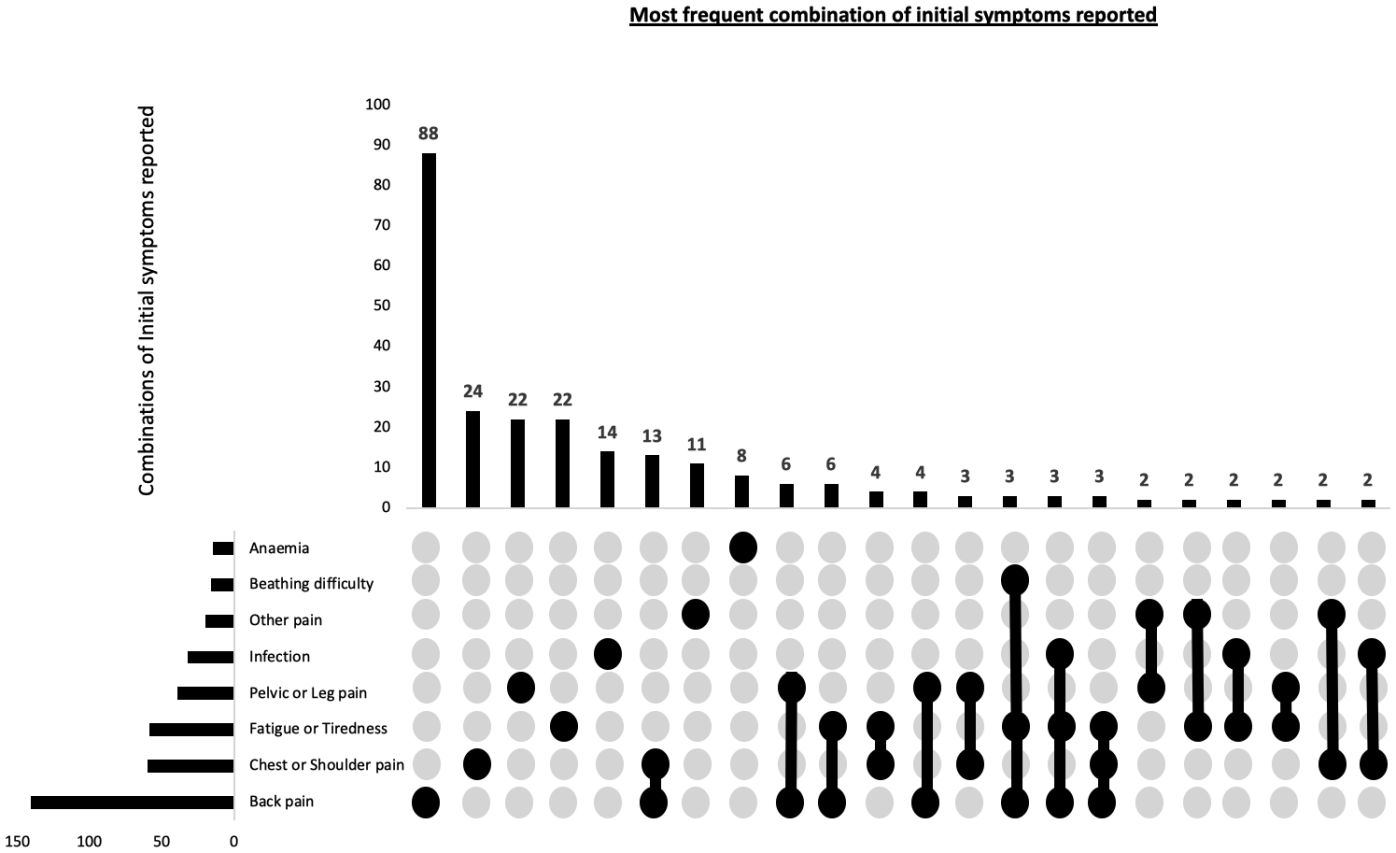
Combination of patient reported potential first presenting symptoms of myeloma in 300 patients. This combinatorial plots describes the most frequent sets of initial symptoms reported by participants and also the frequency of individual symptoms.

Among the self-reported initial symptoms, the most prevalent combination reported by patients was back pain/vertebral fractures, accounting for 47% of the cases. This was followed by chest/shoulder pain, including rib pain and fractures, reported by 20% of the patients, and fatigue/tiredness reported by 19.7% of the patients (**Table 3**). Given that participants may present with multiple initial symptoms, combinatorial analysis was conducted to assess the most frequent symptom combinations. The predominant single symptom was back pain, occurring in isolation, and was reported by 29.3% of all participants. The top three combinations of symptoms were back pain, chest/shoulder pain and pelvic/leg pain (**Figure 1**).

**Table 3:** Types of initial symptoms reported by patients.

Initial symptoms reported by patients	N = 300^1^
Back pain (including vertebral fractures)	141 (47%)
Chest and shoulder pain (including rib fractures)	60 (20%)
Fatigue or tiredness	59 (20%)
Other symptoms^*^	56 (19%)
Pelvic or leg pain	39 (13%)
Infection	32 (11%)
Other pain	20 (6.7%)
Breathing difficulty	16 (5.3%)
Anaemia	15 (5.0%)
Abdominal discomfort	7 (2.3%)
Other fractures	3 (1.0%)
Kidney Disease	3 (1.0%)

^1^n (%).

As more than one symptom could have been self-reported, the percentage totals exceed 100%.

*Refers to self-reported symptoms that are vague and could not be categorised into any other groups.

### Healthcare professionals seen

**Figure 2:**
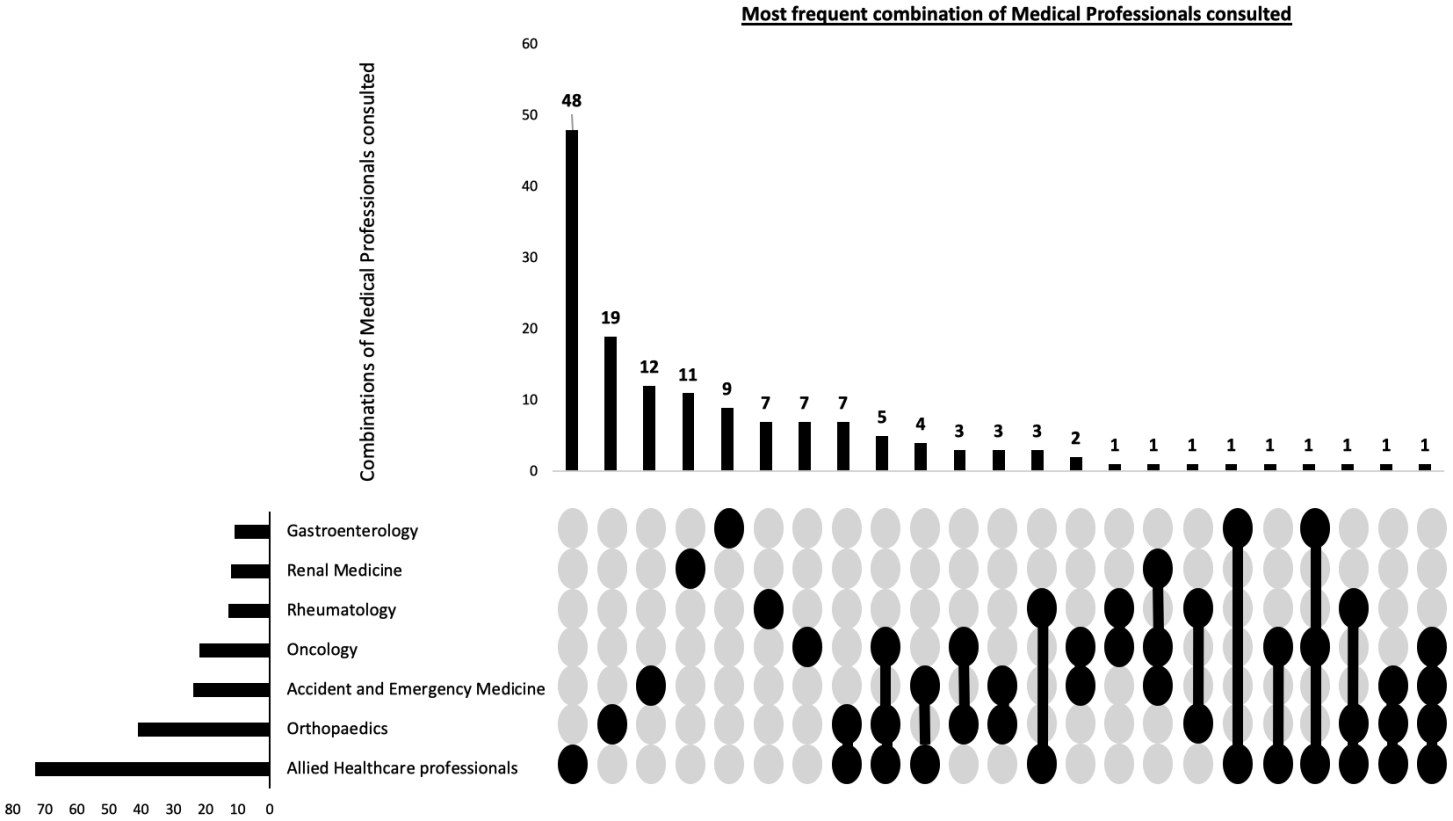
Combination of patient reported healthcare professionals seen prior to myeloma diagnosis. This combinatorial plots describes the most frequent types of healthcare professional seen during the diagnostic journey as well as the frequency of individual specialities.

Of the 300 participants, 120 (40%) of participants were diagnosed by direct referral from primary care to haematology, without seeing any other healthcare professionals. In the remaining 180 participants, other than a GP and a haematologist, the most common healthcare professionals consulting with these patients prior to their diagnosis included AHPs (41%) and Orthopaedic surgeons (23%). **Table 4** summarises the specialities that assessed the patients before their MM diagnosis. Considering that a majority of patients had received interim diagnoses before being diagnosed with MM, it was likely that they had consulted multiple healthcare professionals. Therefore, we conducted a combinatorial analysis to determine the most common combinations of healthcare professionals consulted. The single predominant healthcare professional consulted during their diagnostic journey were the AHPs, with 26.7% of participants not directly referred to haematology consulting them individually. The combinatorial analysis concerning the most frequent combinations of healthcare professionals consulted had the same top 3 most frequent healthcare specialities as individual specialities (**Figure 2**).

**Table 4:** Healthcare professionals consulted on route to final MM diagnosis.

Healthcare professionals seen	N = 180^1^
Allied Healthcare Professionals	73 (41%)
Orthopaedics	41 (23%)
A&E physicians	24 (13%)
Oncologists	22 (12%)
Rheumatology	13 (7.2%)
Renal medicine	12 (6.7%)
Gastroenterology	11 (6.1%)
Respiratory	8 (4.4%)
Neurology or neurosurgery	7 (3.9%)
Cardiology	4 (2.2%)
ENT	4 (2.2%)
Ophthalmology	3 (1.7%)
Urology	3 (1.7%)
Pain clinic	3 (1.7%)
General surgery	2 (1.1%)
Endocrinology	2 (1.1%)
Maxillofacial surgery	2 (1.1%)
Dermatologist	1 (0.6%)
Obstetrics and gynaecology	1 (0.6%)
Infectious disease	1 (0.6%)

^1^n (%).

As more than one healthcare professional could have been consulted, the percentage totals exceed 100%.

### The interval from symptom to the diagnosis of MM

Overall, the median time from symptom onset to final diagnosis was 4 months (IQR 2-10 months, range 0-172). Of the participants, 129 patients (43%) were diagnosed within 12 weeks months of initial symptom onset and 171 patients (57%) were diagnosed more than 12 weeks following symptom onset.

The results of the univariate logistic regression are shown in **Table 5**. In the initial multivariate model, which considered key predictors of diagnosis within 12 weeks, several significant findings emerged regarding factors influencing the diagnostic delay of the disease (shown in **Supplementary Table 1**). The multivariate model following backward elimination included only five predictors (**Table 5**). The single factor that is most strongly independently associated with diagnostic delay in MM was AHP (OR=0.25 [0.12, 0.47], p= <0.001). Infection-related symptoms [OR= 0.32 [0.13, 0.76], p = 0.013) and experiencing chest or shoulder pain (OR= 0.45 [0.23, 0.86], p=0.020) also reduced the likelihood of being diagnosed within 12 weeks, while older age reporting symptoms (OR=1.04 [1.02, 1.07], p= 0.001) independently significantly increased the likelihood of being diagnosed within 12 weeks. Back pain was the only predictor which was selected in the final model that was not statistically significant in predicting diagnosis within 12 weeks (OR= 1.61 [0.96, 2.74], p= 0.08). **Table 5** provides a summary of the results from both the univariate and multivariate analyses.

**Table 5:** Combined univariate and multivariate backward step-wise model for predicting diagnosis of MM within 12 weeks.

Predictor	Univariate			Multivariate Step-wise		
	OR^1^	95% CI^1^	p-value	OR^1^	95% CI^1^	p-value
AHPs	0.28	0.15, 0.52	**<0.0001**	0.25	0.12, 0.47	**<0.001**
Orthopaedics	0.88	0.44, 1.72	0.70	–	–	–
Renal medicine	1.93	0.60, 6.64	0.27	–	–	–
Oncology	0.60	0.22, 1.48	0.29	–	–	–
Gastroenterology	0.49	0.11, 1.73	0.30	–	–	–
Rheumatology	0.83	0.25, 2.55	0.75	–	–	–
Accident and Emergency	1.37	0.60, 3.20	0.46	–	–	–
Infection	0.43	0.17, 0.96	**0.05**	0.32	0.13, 0.76	**0.013**
Fatigue or tiredness	1.38	0.77, 2.46	0.28	–	–	–
Anaemia	1.18	0.40, 3.38	0.75	–	–	–
Breathing difficulty	1.78	0.64, 5.10	0.27	–	–	–
Age at symptom	1.05	1.02, 1.08	**<0.001**	1.04	1.02, 1.07	**0.001**
Female	0.72	0.45, 1.15	0.17	–	–	–
Back pain (including vertebral fractures)	1.29	0.81, 2.04	0.28	1.61	0.96, 2.74	0.08
Chest and shoulder pain (including rib fractures)	0.46	0.24, 0.83	**0.01**	0.45	0.23, 0.86	**0.020**
Other pain	0.89	0.34, 2.21	0.80	–	–	–
Pelvic or leg pain	0.81	0.40, 1.61	0.56	–	–	–

^1^OR, Odds Ratio; CI, Confidence Interval.

Bold indicates p <0.05.

## Discussion

Overall, our findings show that the time interval from the first potential symptom to the final diagnosis of MM is approximately 4 months, that back pain, chest/shoulder pain, and pelvis/leg pain were the most frequently reported initial symptoms and that referral to an AHP was the strongest independent predictor of a long diagnostic interval.

In accordance with previous studies, our single-centre study also identified back pain as the most common initial presenting symptom in patients with multiple myeloma. This observation aligns with several studies that reported an increase in symptoms such as back pain, rib pain, infections, and chest pain approximately 2 years prior to diagnosis. (28, 29) This consistency across studies underscores the potential of these symptoms, particularly back pain, as early indicators of MM.

Our median diagnostic interval from the first symptom to the diagnosis was 4 months in MM. Previous studies have not been consistent with some studies reporting shorter diagnostic intervals (13, 16), similar diagnostic intervals (12) and other studies reporting longer diagnostic intervals in MM. (10, 17) When comparing diagnostic timeframes for different types of cancer, breast cancer had a median diagnostic delay of 115 days, while bladder cancer had 63 days, cervical cancer had 60 days, and colorectal cancer had 26 days. (30) Hence, in our study, we demonstrate a longer symptom-to-diagnosis time than commonly reported cancers.

Regarding the symptom-to-diagnosis interval, most studies do not account for the patient appraisal interval meaning the interval would likely be longer than it should be. Additionally, our study differs from others as it excludes pre-myeloma conditions like MGUS and smouldering myeloma. Conversely, diagnostic intervals measured from healthcare databases usually include patients with smouldering myeloma and MGUS. Moreover, the median age of individuals presenting with MM symptoms in most studies is around 70 years, while in our study, the median age of participants was 67 years. This age difference could lead to symptoms related to MM being misattributed to other age-related diseases, resulting in potential delays in diagnosis. (31) As a result, more than half of the patients experience multiple repeat consultations in primary care with GPs before being referred to a specialist. (32) The non-specific nature of MM symptoms also increases the likelihood of referrals to specialists who are not haematologists, including AHPs such as physiotherapists and osteopaths, further contributing to delays in diagnosis and treatment initiation.

Finally, our prediction tool utilises a combination of five variables: AHP referral, presence of infection, age of symptom onset, chest/shoulder pain, and back pain. This tool demonstrated a reasonable level of performance in identifying individuals who may be at risk of delayed diagnosis of MM, with discrimination that is clinically acceptable. Additionally, in contrast to a previous study that suggested patients with anaemia and back pain were more likely to experience diagnostic delays in MM, our study found that neither back pain or anaemia are significant predictors of diagnostic delay. (13) However, in alignment with a previous study’s findings that indicated younger patients were twice as likely to encounter delays in diagnosis of MM, characterised by having three or more GP consultations prior to referral, we show that older age is a significant predictor for diagnosis within 12 weeks. (32) Overall, before implementing this tool in a clinical setting, it is essential to conduct additional validation using an external dataset.

These findings highlight the need to include chest and shoulder pain as potential early symptoms of MM. (33–35) Another key insight is that the AHPs such as physiotherapists, chiropractors and osteopaths are potential gatekeepers for diagnosis given they are consulted so often during the diagnostic journey to MM diagnosis. Further work is needed to understand if there are specific patient characteristics that could identify these patients amongst musculoskeletal referred patients, e.g. sex, age, type of symptom and response to physical therapy.

Our study has some limitations. A major limitation of this study is that participants had to self-report all aspects of their diagnostic journey which means that there will likely be uncertainty of whether the first reported symptom was clinically related to the MM as well as the exact onset. This could have led to the time interval from symptom onset to final diagnosis being greater than in previous studies in which the median time from symptom onset to final diagnosis was 99 days. (12, 13, 16) Furthermore, while efforts were made to be as inclusive as possible to capture a broad spectrum of MM patients, it is acknowledged that the study may have primarily captured a subset of patients who are computer-literate and well enough to complete an online questionnaire. This could have potentially skewed the data towards younger patients and those with less advanced or aggressive disease. Furthermore, very ill patients or those who experienced early death may not have been accounted for, which could be associated with later diagnosis and advanced-staged disease. This is a limitation that needs to be considered when interpreting the results. The usability of the online forms was tested and iteratively improved based on user feedback to ensure as many people as possible could complete them. However, there may still be barriers for some individuals, and future work could explore alternative methods of data collection to increase inclusivity. Another limitation is that components to contextualise the overall journey to a cancer diagnosis were not recorded. Hence, definitive conclusions regarding the time intervals between the first consultation with a GP and the final diagnosis of MM, as well as the time intervals between the first hospital consultation and the final diagnosis of MM, could not be drawn. This limitation also arises from the presence of recall bias, as many participants in the cohort were unable to accurately recall the precise dates of their initial GP or hospital consultations for symptoms consistent with MM.

Future studies could link patient-reported information with GP or hospital records. This approach could have enabled the study to assess if the time span between the final MM diagnosis and the GP consultation meets the current objectives of excluding cancer or confirming a diagnosis within 28 days. However, we recognise that due to the age of the MM population and their associated co-morbidities (which were not investigated in this study), it would remain a significant challenge to discern their initial visit related to MM and their first set of MM symptoms. Additional risk factors that could have predicted delayed diagnosis of MM which was not captured in our prediction model include the geographical place of diagnosis, Indices of Deprivation and the effect of the COVID-19 pandemic. The lack of specificity of hospital names by patients meant that these data could not be included as one name could link to more than one hospital. Finally, we recognise that given less than 50% of RUDY MM patients completed the diagnostic form may have biased the findings.

In conclusion, our findings indicate that developing resources for AHPs could potentially enhance early detection of MM, given its substantial impact in reducing the likelihood of diagnosing MM within a 12-weeks. This could include creating standardised guidelines or protocols for AHPs to encourage them to consider and screen for the diagnosis, and hence reduce diagnostic delay of MM.

## Data availability statement

The raw data supporting the conclusions of this article will be made available by the authors, without undue reservation.

## Ethics statement

The studies involving humans were approved by South Central Research Ethics Committee (LREC 14/SC/0126 & RUDY LREC 17/SC/0501). The studies were conducted in accordance with the local legislation and institutional requirements. The participants provided their written informed consent to participate in this study.

## Author contributions

RV: Data curation, Formal Analysis, Investigation, Software, Writing – original draft, Writing – review & editing. KS: Data curation, Formal Analysis, Investigation, Software, Writing – original draft, Writing – review & editing. RS: Data curation, Formal Analysis, Investigation, Software, Supervision, Writing – review & editing. SB: Supervision, Writing – review & editing. MS: Data curation, Formal Analysis, Investigation, Software, Supervision, Writing – review & editing. KR: Supervision, Writing – review & editing. MJ: Conceptualization, Data curation, Formal Analysis, Funding acquisition, Investigation, Methodology, Project administration, Software, Supervision, Writing – original draft, Writing – review & editing.
