# Supplementary material for: Patient-reported symptoms and diagnostic journey in Multiple Myeloma
Source: Front Oncol. 2023 Nov 30;13:1282569. doi: 10.3389/fonc.2023.1282569 (PMC10720586; doi:10.3389/fonc.2023.1282569)
Supplement: Supplementary file 1 [file Table_1.docx]

Supplementary Table 1: **Initial full multivariate logistic regression model (prior to backward stepwise model)** for predicting MM diagnosis within 3months:

| **Predictor** | **OR**^1^ | **95% CI**^1^ | **p-value** |
| --- | --- | --- | --- |
| AHPs | 0.26 | 0.13, 0.50 | **<0.001** |
| Orthopaedics | 1.14 | 0.50, 2.57 | 0.8 |
| Renal medicine | 1.62 | 0.45, 6.25 | 0.5 |
| Oncology | 0.48 | 0.16, 1.36 | 0.2 |
| Gastroenterology | 0.52 | 0.10, 2.09 | 0.4 |
| Rheumatology | 1.38 | 0.35, 5.21 | 0.6 |
| A&E physicians | 1.67 | 0.64, 4.42 | 0.3 |
| Infection | 0.31 | 0.12, 0.77 | **0.016** |
| Fatigue or tiredness | 1.51 | 0.76, 2.97 | 0.2 |
| Anaemia | 1.08 | 0.33, 3.42 | >0.9 |
| Breathing difficulties | 1.65 | 0.53, 5.38 | 0.4 |
| Age at first symptom | 1.04 | 1.02, 1.07 | **0.002** |
| Female | 0.73 | 0.42, 1.27 | 0.3 |
| Back pain (including vertebral fractures) | 1.63 | 0.91, 2.96 | 0.10 |
| Chest and shoulder pain (including rib fractures) | 0.44 | 0.21, 0.85 | **0.018** |
| Other pain | 0.87 | 0.29, 2.50 | 0.8 |
| Pelvic or leg pain | 0.97 | 0.43, 2.16 | >0.9 |
| ^1^OR = Odds Ratio, CI = Confidence Interval | | | |


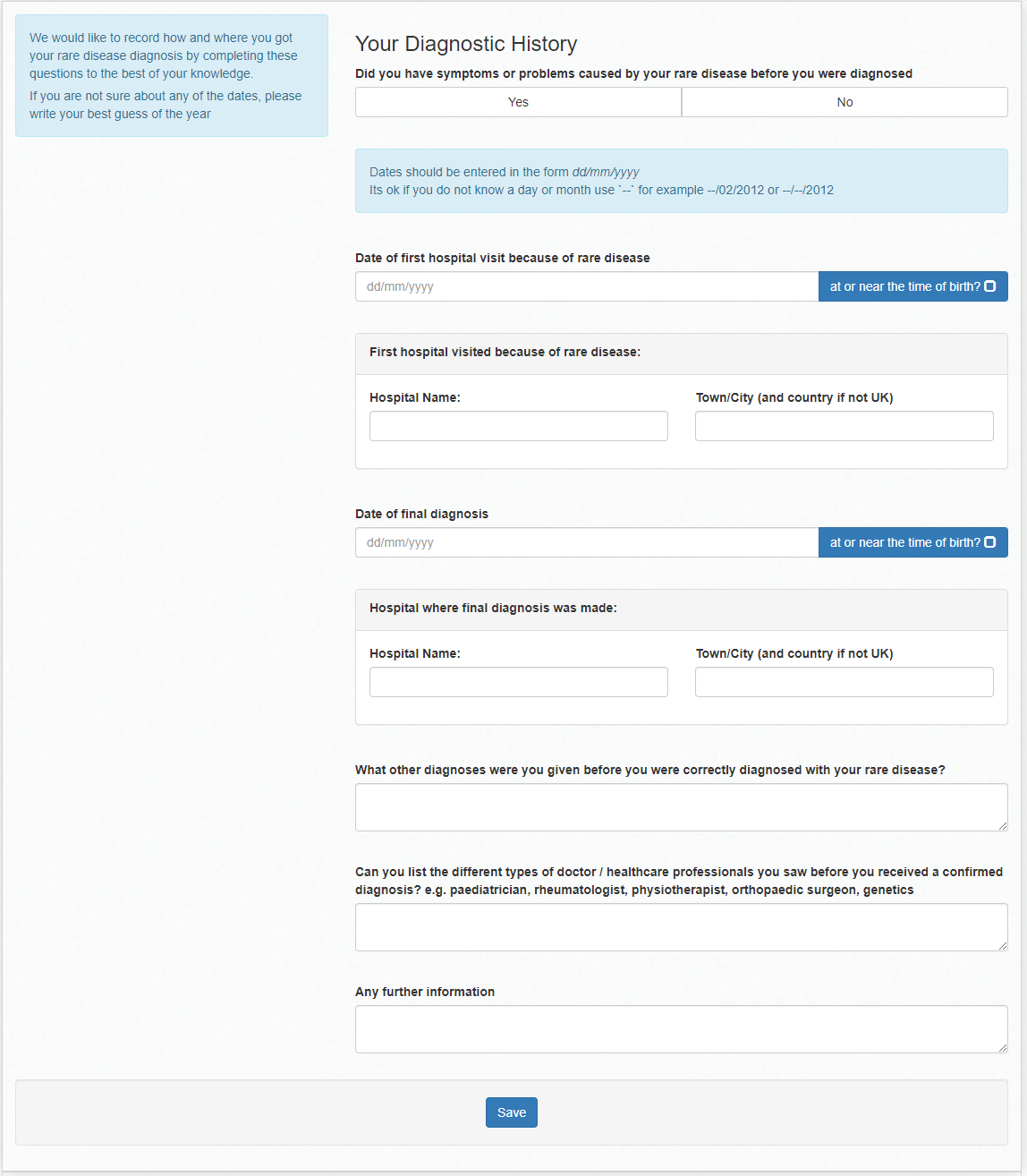
Supplementary questionnaire 1: RUDY Study Questionnaire
